# Characterization of *Sucrose transporter *alleles and their association with seed yield-related traits in *Brassica napus *L

**DOI:** 10.1186/1471-2229-11-168

**Published:** 2011-11-23

**Authors:** Fupeng Li, Chaozhi Ma, Xia Wang, Changbin Gao, Jianfeng Zhang, Yuanyuan Wang, Na Cong, Xinghua Li, Jing Wen, Bin Yi, Jinxiong Shen, Jinxing Tu, Tingdong Fu

**Affiliations:** 1 National Key Laboratory of Crop Genetic Improvement, National Center of Rapeseed Improvement in Wuhan, Huazhong Agricultural University, Wuhan 430070, China

## Abstract

**Background:**

Sucrose is the primary photosynthesis product and the principal translocating form within higher plants. *Sucrose transporters *(*SUC/SUT*) play a critical role in phloem loading and unloading. Photoassimilate transport is a major limiting factor for seed yield. Our previous research demonstrated that *SUT *co-localizes with yield-related quantitative trait loci. This paper reports the isolation of *BnA7.SUT1 *alleles and their promoters and their association with yield-related traits.

**Results:**

Two novel *BnA7.SUT1 *genes were isolated from *B. napus *lines 'Eagle' and 'S-1300' and designated as *BnA7.SUT1.a *and *BnA7.SUT1.b*, respectively. The BnA7.SUT1 protein exhibited typical SUT features and showed high amino acid homology with related species. Promoters of *BnA7.SUT1.a *and *BnA7.SUT1.b *were also isolated and classified as *pBnA7.SUT1.a *and *pBnA7.SUT1.b*, respectively. Four dominant sequence-characterized amplified region markers were developed to distinguish *BnA7.SUT1.a *and *BnA7.SUT1.b*. The two genes were estimated as alleles with two segregating populations (F_2 _and BC_1_) obtained by crossing '3715'×'3769'. *BnA7.SUT1 *was mapped to the A7 linkage group of the TN doubled haploid population. *In silico *analysis of 55 segmental *BnA7.SUT1 *alleles resulted three *BnA7.SUT1 *clusters: *pBnA7.SUT1.a- BnA7.SUT1.a *(type I), *pBnA7.SUT1.b- BnA7.SUT1.a *(type II), and *pBnA7.SUT1.b- BnA7.SUT1.b *(type III). Association analysis with a diverse panel of 55 rapeseed lines identified single nucleotide polymorphisms (SNPs) in promoter and coding domain sequences of *BnA7.SUT1 *that were significantly associated with one of three yield-related traits: number of effective first branches (EFB), siliques per plant (SP), and seed weight (n = 1000) (TSW) across all four environments examined. SNPs at other *BnA7.SUT1 *sites were also significantly associated with at least one of six yield-related traits: EFB, SP, number of seeds per silique, seed yield per plant, block yield, and TSW. Expression levels varied over various tissue/organs at the seed-filling stage, and *BnA7.SUT1 *expression positively correlated with EFB and TSW.

**Conclusions:**

Sequence, mapping, association, and expression analyses collectively showed significant diversity between the two *BnA7.SUT1 *alleles, which control some of the phenotypic variation for branch number and seed weight in *B. napus *consistent with expression levels. The associations between allelic variation and yield-related traits may facilitate selection of better genotypes in breeding.

## Background

Sucrose is the principal transport form of photosynthetically assimilated carbohydrate in higher plants. It is synthesized in the source leaf or the pericarp of the pod and transported via the phloem to sink tissues and provides energy and carbon skeleton to the non-photosynthetic tissues. In sink tissues, sucrose may be used directly for metabolism or translocated to storage tissues (such as cotyledon and endosperm) for synthesis of three major storage products (oil, starch, and protein) through carbohydrate metabolism. On the basis of these storage products, crops are designated as oleaginous, farinose, or proteinacious crops [1-4].

Sucrose transporter (SUT) was first reported in spinach (*Spinacia oleracea *L.) (Amaranthaceae) [5]. In the last two decades, cDNA for SUTs has been isolated and cloned in higher plants (e.g., Solanaceae, Brassicaceae, Amaranthaceae, Poaceae) [6-8]. Immunolocalization analysis revealed that SUTs are located in plasma membranes of enucleate sieve and companion cells [9,10]. *SUTs *have been reported to be expressed in various tissues of the transport pathway and sink cells in *Arabidopsis*, barley, potato, and rubber [9-13]. Mutation studies of *SUTs *have revealed that SUTs are responsible for restraining plant growth and pollen germination [14-16]. Antisense transformation experiments have clearly shown that SUTs also are responsible for retardation of sucrose translocation, fruit size reduction, and lowered fertility in tomato [17,18]. Overexpression transformations showed lower sucrose concentration in leaves and increased growth rates of pea cotyledon [19,20]. Early stages of seed development in *Brassica *exhibit a SUT association with starch and oil accumulation in the embryo; the further growth of the cotyledon leads to lipid synthesis and starch degradation [2,21]. Results from another study have suggested that increased lipid synthesis is an effect of sucrose unloading [22]. However, detailed reports are lacking for *SUT *in *Brassica napus *(Brassicaceae).

*B. napus *is one of the major global oil crops. It is used for direct human consumption, as animal feed, and recently as a source of bio-fuel. High seed yield per unit is one of the most important challenges in *B. napus *breeding, while the harvest index (HI) is only about 0.2-0.3 [23,24]. Generally, the HI of cereal crops can reach 0.5-0.6 in crop production under suitable conditions and management, with reserved assimilates in plants contributing 10-40% of the final yield at the grain filling stage [25]. The HI of soybean, one of the most important oil crops, also can reach 0.4-0.6 [26,27] and has been successfully maximized during breeding [28]. Investigations have indicated that source and sink organs are not limiting, while assimilate translocation is the most critical limiting factor for seed yield in *Brassica *[29,30]. *SUT *may be a key gene for increasing seed yield by translocating sucrose from source to sink.

In our previous investigation, a functional marker derived from SUT was co-localized with seed yield quantitative trait loci (QTLs) in *B. napus *[31]. We hypothesized that the *SUT *gene affects seed yield in *B. napus*. Here, a complete *SUT *(*BnA7.SUT1*) and promoters were isolated and characterized. A series of experiments and observations of the *B. napus SUT *made it possible to detect alleles located in the A7 linkage group, and allelic variation of *BnA7.SUT1 *was associated with seed yield-related traits. *BnA7.SUT1.b *and its promoter are linked to higher seed yield, while *BnA7.SUT1.a *is associated with increased seed weight.

## Results

### Isolation of *BnA7.SUT1*

Three *Brassica *fragments (two expressed sequence tagged and a bacterial artificial chromosome [BAC]; respective GenBank accession numbers AY190281, AY065839, and AC189334) were obtained from the large-scale sequence analysis results at The Arabidopsis Information Resource database and identified as having high sequence homology with the *Arabidopsis AtSUC1 *(At1G71880) sequence [6]. Primers (M1-M4) were designed based on conservative segments (see Additional file 1). With these primers, the main genomic segments of *BnX.SUT1 *were generated; the remnant fragments and promoter were obtained by thermal asymmetric interlaced (TAIL) PCR in the *B. napus *cultivar 'Eagle'. According to the contig, the complete open reading frame (ORF) was identified by using gene prediction programs (GENSCAN; FGENESH), and gene-specific primers were developed to generate *BnX.SUT1 *in line 'S-1300'. Of interest, the PT1 primer pair, which amplifies the 5'-end of *BnX.SUT1*, generated the expected band in 'Eagle' exclusively (Table 1). Thus, more than 2 kb of promoter and 5' untranslated region (UTR) were obtained by TAIL-PCR from 'S-1300', respectively. Based on the predicted 5' and 3' UTRs of the candidate *SUT*-like gene, common gene-specific primers were designed: sut-2L (5'-AGA ATG GGA GCT TTT GAA ACA G-3') and sut-2R (5'-GGC ATA GAG TAC ACT AAT GGA AG-3'). These primers were used to amplify the full-length cDNA and genomic sequences of *BnX.SUT1*. Forty-four cDNA sequences were isolated from various organs/tissues of 'Eagle' and 'S-1300' and were classified into four clusters (Additional file 2). Two clusters showed non-variation sequences and non-distinguished expression in six *B. napus *lines (data not shown) and were not included in further work in this investigation. The other two clusters were designated as *BnA7.SUT1.a *and *BnA7.SUT1.b*, obtained from 'Eagle' and 'S-1300', respectively.

**Table 1:** Details of SCAR markers from *BnA7.SUT1 *showing significant associations (*P *value) with yield-related traits in the set of 55 genetically diverse *Brassica napus *genotypes

Symbol	Primer	Primer sequence	Length	Product(bp)
	name		Eagle	S-1300
PT1	PT1-L	ATGTTCGCTGGCATACCTAG	1600	--
	PT1-R	TTCCGACCAATCCACTCAAC		
PT5	PT5-L	ATATACAGCATGAACGCAAC	--	600
	PT5-R	ATGAGAGAGGACCATTTGTG		
ET3	ET3-L	GTTGTAGAGACACAGCCACCTTC	1250	--
	ET3-R	CGGCAGTTTTCCGGTGAC		
ET4	ET4-L	GTTGTAGAGACACAGCCACCTTC	--	850
	ET4-R	TTCGTCGCCGGAGTTTGG		

**Figure 1:**
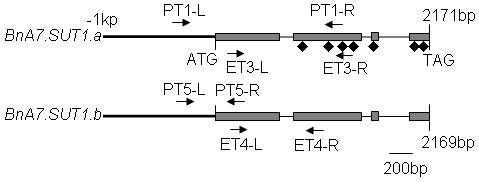
**Comparison of the nucleotide sequences of *BnA7.SUT1.a *and *BnA7.SUT1.b***. The *gray bars *and *black lines *denote exons and introns in the CDS, while *thick black lines *indicate the promoter of *BnA7.SUT1*. The *black rhombus *and *arrowhead with names *distinguish amino acid sites and the locations of primers, respectively.

Both putative ORFs of *BnA7.SUT1.a *and *BnA7.SUT1.b *contain 1545 bp and encode a protein of 514 amino acids. The combination of the cDNA and genomic DNA sequences revealed that the *BnA7.SUT1 *gene is 2593 bp in length, containing four exons and three introns. The hydrophobicity profile analysis of *BnA7.SUT1 *revealed the presence of 12 transmembrane spanning domains, arranged in two sets of six putative transmembrane domains separated by a long central hydrophilic loop, with both terminal domains and a large central loop located on the intracellular side of the plasma membrane. *BnA7.SUT1 *belongs to the subgroup SUT1 (Additional file 3). The two predicted protein sequences are 98% identical, having seven amino acid differences between BnA7.SUT1.a and BnA7.SUT1.b (Figure 1), none of them in transmembrane domains. The cDNA of *BnA7.SUT1 *shared 76% sequence identity with a published *BnSUT *(GenBank accession no. EU570076), which has 508 amino acids. The *BnA7.SUT1 *sequence is very similar to the homologues from related species and showed more than 85% sequence similarity with *AtSUC1 *(AT1G71880) and *BoSUC1 *(AY065839) and 81% sequence similarity with *AtSUC5 *(NM_105847). Hence, the isolated *BnA7.SUT1 *alleles, homologous with *Arabidopsis *and *B. oleracea*, are novel SUT genes in *B. napus*.

### Nucleotide sequence analysis

Seventeen primer pairs were designed to generate fragments of about 400 bp to 1700 bp. Ten primer pairs were designed from the sequences of *BnA7.SUT1.a *and seven from the diverse domains of *BnA7.SUT1.b*. Four markers (Table 1 Figure 1) showed polymorphisms between 'Eagle' and 'S-1300'. ET3 and PT1, which were developed from *BnA7.SUT1.a *and its promoter, generated the expected fragments in 'Eagle' but not in 'S-1300'. By contrast, ET4 and PT5, amplifying *BnA7.SUT1.b *and its promoter, generated the expected bands in 'S-1300' exclusively (Figure 1). The four sequence-characterized amplified region (SCAR) markers were used to analyze the 55 cultivars/lines. And the panel lines were distinguished as three groups by these markers.

The 55 partial *BnA7.SUT1 *genomic fragments of ~1570 bp were amplified from the panel lines using primer pairs PT1-L/PT1-R and PT5-L/PT1-R (Figure 1), which are located 382 bp upstream and 1191 bp downstream from the start codon of *BnA7.SUT1*. In total, 142 single nucleotide polymorphism (SNP) sites were detected among the lines, including 120 SNPs in the promoter and 5'-UTR, 12 SNPs in exons, and 10 in introns. The genetic diversity between two regions was analyzed according to distinct different diversities in the 5'-end and gene regions. Nucleotide diversity was lower in gene regions (π = 0.00534) compared with 5'-end regions (π = 0.13502). Tajima's *D *of gene regions indicated non-significance, while the 5'-end of *BnA7.SUT1 *had a positive and significant Tajima's *D *value (Table 2). The results indicated that selection was present at the 5'-end and that the selection effect had not extended to the entire gene.

**Table 2:** Nucleotide diversity and Tajima's test of *BnA7.SUT1*

Region	Size(bp)	**H**^**b**^	π	Tajima's *D*
5'-end	382	3	0.13502	3.39254**
gene	1194	2	0.00534	1.03602
total	1576	5	0.03586	2.91072**
ba^a^	382	4	0.00178	-1.10746
bb^a^	382	0	0	NA^c^
aa^a^	382	1	0.00034	-1.13284

**, *P *< 0.01

^a ^ba, bb, aa represent 5'-ends of genotypes *pBnA7.SUT1.b-BnA7.SUT1.a*, *pBnA7.SUT1.b-BnA7.SUT1.b*, and *pBnA7.SUT1.a-BnA7.SUT1.a*, respectively

^b ^Number haplotypes, number in cell, ba, bb, and aa represent total polymorphism sites of each genotype.

^c ^NA, not available

**Figure 2:**
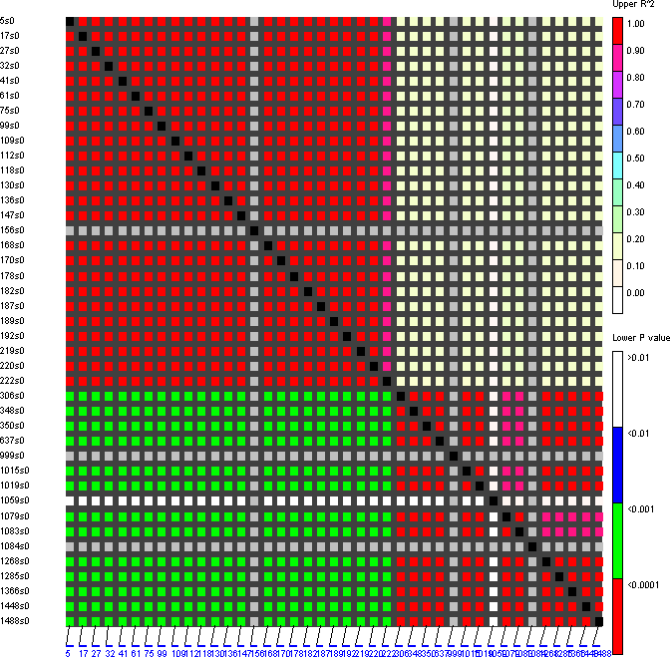
**LD across the 51 *BnA7.SUT1 *loci**. Positions of polymorphisms in the alignment are given. Positions 138, 357, 999, and 1085 are indel polymorphisms, and the remaining polymorphisms are SNPs.

**Figure 3:**
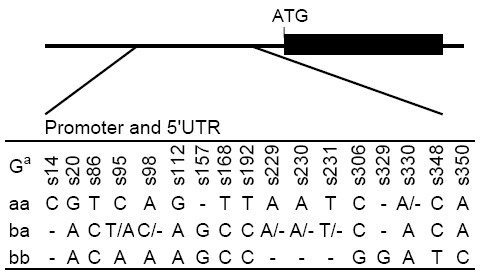
**Sequence comparison of three genotypes in the promoter and 5'UTR of *BnA7.SUT1***. ^a ^G, genotype; aa, ba, bb represent the 5'-ends of *pBnA7.SUT1.a- BnA7.SUT1.a*, *pBnA7.SUT1.b- BnA7.SUT1.a*, and *pBnA7.SUT1.b- BnA7.SUT1.b*, respectively.

Linkage disequilibrium (LD) was estimated between 51 pairs of polymorphic sites (SNPs and indels) in the *BnA7.SUT1 *sequence; two LD blocks were observed at the 5'-end and gene regions, respectively (Figure 2). Abundant SNPs resulted in the same haplotypes among the lines, which could be classified into three clusters consistent with the results of the neighbor-joining distance tree (Additional file 4). Overall, we found interesting results indicating that the *BnA7.SUT1.a *promoter regulates only *BnA7.SUT1.a *and that the *BnA7.SUT1.b *promoter regulates both *BnA7.SUT1.a *and *BnA7.SUT1.b*, designated as *pBnA7.SUT1.a- BnA7.SUT1.a *(type I), *pBnA7.SUT1.b- BnA7.SUT1.a *(type II), and *pBnA7.SUT1.b- BnA7.SUT1.b *(type III). Nucleotide diversity was also separately evaluated for type I, type II, and type III based on 382-bp sequences of the 5'-end (Table 2). Type III and type I presented no polymorphisms and one indel (s330) among 18 and 16 lines, respectively. However, type II showed one SNP (s95) and three indels (s98, s229, and s330) among 21 lines (Figure 3).

### Allelism analysis and genetic mapping

To identify the allelism of *BnA7.SUT1.a *and *BnA7.SUT1.b*, '3715' (type III) and '3769' (type I) were used to develop F_1_, F_2_, and BC_1 _populations. With these populations, segregation ratios were analyzed using the markers PT1 and PT5. The segregation of heterozygous F_1 _plants to single-band plants in the F_2 _population showed the expected Mendelian ratio of 1:2:1 (number of plants was 45:119:61) (χ^2 ^= 3.03, 0.10 <*P *< 0.25), and the expected ratio of 1:1 (number of plants was 46:49) in the BC_1 _population (χ^2 ^= 0.074, 0.75 <*P *< 0.90). Therefore, *BnA7.SUT1.a *and *BnA7.SUT1.b *appeared to be alleles at a single locus.

**Figure 4:**
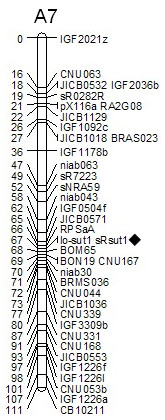
**Mapping of *BnA7.SUT1 *in the TN DH linkage map**. The marker sRsut1 was derived from the BAC containing *BnA7.SUT1*.

*BnA7.SUT1 *showed high similarity with the BAC (AC189334) from *B. rapa*, and three simple sequence repeats (SSR) markers were developed according to the BAC sequence. Additionally, gene-specific primers were designed based on *BnA7.SUT1.b*. An SSR marker (sRsut1) and a gene-specific marker (lo-sut1) showed the same polymorphisms found in the 'Tapidor-NY7' (TN) doubled haploid (DH) population. Hence, *BnA7.SUT1 *was mapped to the A7 linkage group of the TN DH genetic map (Figure 4), consistent with result of Li et al. [31].

### Association of *BnA7.SUT1 *with yield-related traits

The mean phenotypic values for individual lines across four environments ranged from 4.1 to 9.8 for the number of effective first branches (EFB); 111.6 to 312.2 for the number of siliques per plant (SP); 11.5 to 32.5 for the number of seeds per siliques (SS); 5.0 to 19.5 g for seed yield per plant (PY); 174.0 to 842.1 g for block yield (BY); and 2.2 to 5.4 g for seed weight (TSW) (n = 1000), respectively. Analysis of variance showed significant (*P *= 0.01) phenotypic variation for all six yield-related traits among the 55 lines (Table 3), indicating that the assembled panel is suitable for association analysis. Heritabilities were 83.7%, 82.4%, 91.4%, 62.6%, 78.7.6%, and 95.1% for EFB, SP, SS, PY, BY, and TSW, respectively (Table 3). Significant positive phenotypic and genetic correlations between EFB and SP and between yield and silique traits (SP and SS) were observed (Table 3), indicating that an increase in any of the EFB, SP, or SS traits can increase seed yield.

**Table 3:** Descriptive statistics, variance components, correlation coefficients, and heritability for six yield-related traits

Category		EFB	SP	SS	PY(g)	BY(g)	TSW (g)
Descriptive satistics	Range	4.1-9.7	111.6-312.2	11.5-32.5	5.0-19.5	174.0-842.0	2.2-5.4
	Mean ± SD	6.5 ± 0.8	196.6 ± 30.7	20.0 ± 3.3	10.8 ± 1.3	474.0 ± 75.5	3.7 ± 0.5
Variance components	Genotype	7.6**	5.4**	16.5**	3.2**	5.5**	33.4**
	Envrionment	7.3**	26.6**	5.3**	106.5**	64.8**	7.9**
	G×E	1.3*	0.9	1.5**	1.3*	1.3*	1.7**
Correlation coefficients^a^	EFB		0.74**	-0.30*	0.05	0.13	-0.26*
	SP	0.71**		-0.54**	0.20	0.27*	-0.21
	SS	-0.25	-0.45**		0.17	0.28*	-0.48**
	PY(g)	0.06	0.34*	0.14		0.79**	-0.05
	BY(g)	0.17	0.3*	0.22	0.69**		-0.07
	TSW (g)	-0.24	-0.18	-0.46**	-0.01	-0.04	
Heritability%		83.70	82.40	91.40	62.60	78.70	95.10

EFB, number of effective first branches; SP, number of siliques per plant; SS, number of seeds per siliques; PY, seed yield per plant; BY, block yield; TSW, seed weight (n = 1000); **, *P *< 0.01; *, *P *< 0.05

^a ^The numbers above the diagonal are genetic correlation coefficients, and the numbers below the diagonal are phenotypic correlation coefficients.

**Figure 5:**
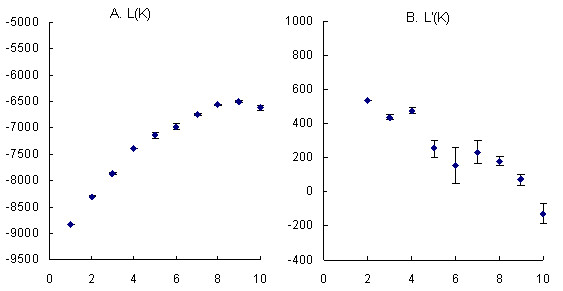
**Estimation of the most appropriate group K values, calculation SD over 10 independent runs**. Mean L(K) over 10 runs for each K value. (B) Change rate of likelihood value calculated as L'(K) = L(K) -L(K-1). We followed the method of Evanno et al. [64].

**Figure 6:**
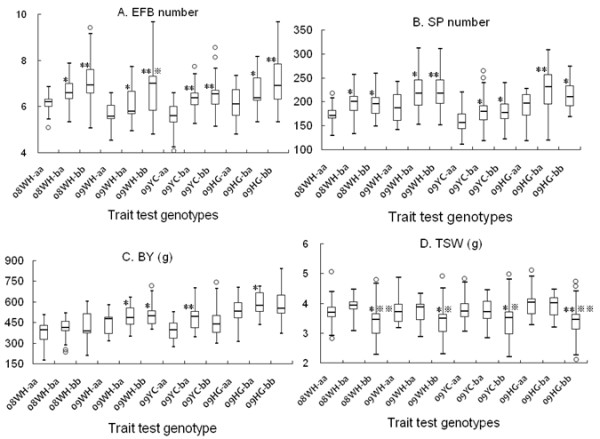
**Box-plots showing distributions of EFB, SP, BY, and TSW within types I, II, and III**. (A) (B) (C) (D) are phenotypic details for EFB, SP, BY, and TSW, respectively. 08WH, year 2008 Wuhan; 09WH, year 2009 Wuhan; 09YC, year 2009 Yichang; 09HG, year 2009 Huanggang. aa represents genotype *pBnA7.SUT1.a- BnA7.SUT1.a*; ba represents genotype *pBnA7.SUT1.b- BnA7.SUT1.a*; and bb represents genotype *pBnA7.SUT1.b- BnA7.SUT1.b*. * t-test between ba genotype and aa genotype, between bb genotype and aa genotype significant at *P *= 0.05, ** significant at *P *= 0.01; ※ t-test between bb genotype and ba genotype significant at *P *= 0.05, ※ ※ significant at *P *= 0.01.

The panel lines evaluated for yield-related traits were mostly modern cultivars and breeding materials. There were considerable differences among the panel lines according to UPGMA cluster (Additional file 5). Population structure was observed among the 55 cultivars/lines based on the method by Hasan et al. [32].The slope of average likelihoods for the overall population was modeled at *K *= 4 (Figure 5); the most stable prediction (standard deviation = 1.99) was obtained with four groups. Each group consisted of 18, 16, 16, and 5 oilseed lines, respectively. Taking the LD (r^2 ^> 0.8) level among sites into account and eliminating same-haplotype SNPs, five sites were significantly associated with at least one of the six yield-related traits (P < 0.05). Information including location, genotype, frequency, and probability value for each site is shown in Table 4. Of interest, the SNP sites (s60 and s222) from the promoter and s1448 from the exon of *BnA7.SUT1 *were associated with EFB and TSW and explained an average 12% and 11% of phenotypic variation throughout the four environments, respectively. The s222 SNP at the promoter in turn affected SP (Table 4). Phenotypic distributions of the previous four yield-related traits are illustrated in three genotypes by box-plots in Figure 6. Promoter *BnA7.SUT1.b *was associated with an increased EFB number and SP number. For TSW, no significant differences were observed between type I and type II with *BnA7.SUT1.a*. However, a significant difference was observed between type I and type III and between type II and type III with different *BnA7.SUT1 *alleles. Hence, polymorphisms at the promoter and coding domain sequence (CDS) of *BnA7.SUT1 *affect yield-related traits interactively.

**Table 4:** Associations among six yield-related traits and the polymorphisms of BnA7.SUT1

Site	Loc	G	F	Env	**EFB**^**a**^	**SP**^**a**^	**SS**^**a**^	**PY**^**a**^	**BY**^**a**^	**TSW**^**a**^
s60	Pro	T/A	16/39	08WH	0.0145					
				09WH	0.0446	0.0374		0.0358	0.0419	
				09YC	0.0016				0.0392	
				09HG	0.0192	0.0190				0.0120
s95	Pro	C/A	16/34	08WH	0.0158		0.0465			
		/T	/5	09WH						
				09YC	0.0058		0.0345			
				09HG		0.0391				0.0401
s222	Pro	A/T	15/40	08WH	0.0098	0.1005				
				09WH	0.0482	0.0255		0.0444	0.0440	
				09YC	0.0010	0.0170				0.0355
				09HG	0.0397	0.0197				0.0067
s1083	Intron	A/C	36/19	08WH	0.0121		0.0481			0.0009
				09WH	0.0272					0.0076
				09YC						0.0104
				09HG						0.0004
S1448	Exon	C/T	37/18	08WH	0.0090					0.0028
				09WH	0.0088		0.0430			0.0269
				09YC						0.0276
				09HG	0.0209					0.0009

^a ^Significant probabilities

Loc, Location; Pro, Promoter; G, Genotype; F, Frequency; Env, environment; 08WH, year 2008 Wuhan; 09WH, year 2009 Wuhan; 09YC, year 2009 Yichang; 09HG, year 2009 Huanggang

### Expression pattern analysis by real-time PCR

**Figure 7:**
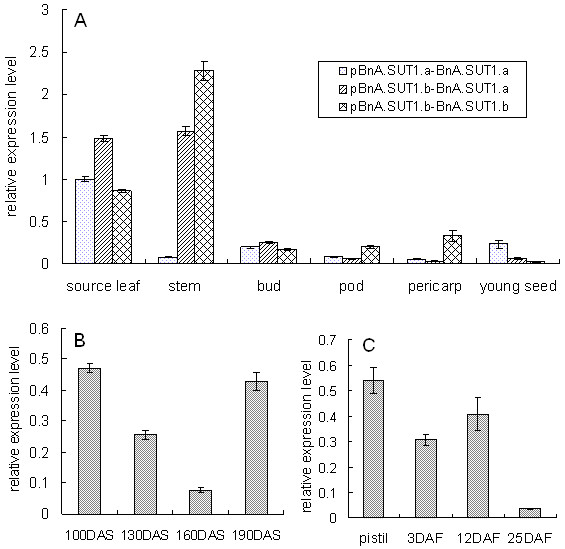
**Real-time PCR analysis of *BnA7.SUT1 *expression**. (A) Expression of *BnA7.SUT1 *in different organs, including source leaf and stem, bud, pod 25 DAF, pericarp of pod, and young seeds among diverse genotypes. (B) Expression in leaf including various developmental stages. 70 DAS is the reproductive stage of winter *B. napus*. (C) Expression in the pod during the developmental period. Pistil was dissected from the bud.

Spatial and developmental expression profiling of *BnA7.SUT1 *was performed using real-time PCR on all three genotypes, type I, type II, and type III, to extract RNA from different organs/tissues at the seed-filling stage. *BnA7.SUT1 *mRNA showed a higher expression level in vegetative organs, reaching its highest level in stems and leaf blades (Figure 7A). Greater abundance was detected in stems of type II and type III genotypes, indicating that the effect of the *BnA7.SUT1.b *promoter was stronger than *BnA7.SUT1.a *promoter in stems. On the other hand, *BnA7.SUT1 *showed lower expression levels in reproductive organs. In flower buds, *BnA7.SUT1 *showed similar transcript levels in all three genotypes.

When pods reached 25 days after flowing (DAF), transcription of *BnA7.SUT1 *showed variations in pods, pericarp, and young seeds in different genotypes. Higher abundance was detected in pods and pericarp of type III genotypes and in developing seeds of type I genotypes. The expression level of *BnA7.SUT1.b *was 3-fold higher than that of *BnA7.SUT1.a *in pods, and 10-fold higher in pericarp. However, the expression of *BnA7.SUT1.b *decreased by three times in developing seeds as compared to *BnA7.SUT1.a*, which showed that *BnA7.SUT1.b *accumulated at higher levels in pods and pericarp, while *BnA7.SUT1.a *produced higher expression levels when regulated by the promoter of *BnA7.SUT1.a *(Figure 7A). Different alleles of *BnA7.SUT1 *also exhibited diverse expression levels when regulated by the same promoter of *BnA7.SUT1.b*, indicating that alleles of *BnA7.SUT1 *also present different gene expression patterns.

In different developmental phases, source leaves were sampled 100 days after sowing (DAS) initially, then at monthly intervals. The abundance of the *BnA7.SUT1 *transcript declined to a low level at flowering. As pods matured, the expression of *BnA7.SUT1 *again showed increased expression levels (Figure 7B). In the developing pods, *BnA7.SUT1 *was highly expressed in the pistil when pods were rapidly elongating and remained at a relatively high level at 3 DAF and 12 DAF. However, expression decreased to a low level at 25 DAF, when the dry weight of pods reached the maximum (Figure 7C).

## Discussion

### Isolation and genetic variations of *BnA7.SUT1*

Our current research describes the isolation of a novel SUT gene *BnA7.SUT1 *and its two alleles *BnA7.SUT1.a *and *BnA7.SUT1.b *in *B. napus*. The cDNAs of *BnA7.SUT1.a *and *BnA7.SUT1.b *showed 18 polymorphic sites and variations in seven amino acids, none of which are located in the SUT transmembrane. The predicted proteins showed similarity with all the other SUTs in amino acid sequence and protein secondary structures with histidine residue position 65 [6,33]. A higher similarity of BnA7.SUT1 with other functional SUTs indicated that BnA7.SUT1 proteins may play an important role in sucrose transport.

We observed frequent sequence exchanges in *BnA7.SUT1 *that resulted in generation of two alleles of *BnA7.SUT1*. Gene conversion and unequal crossing-over are important in generating variation at gene sequences, and recombination events produce novel genotypes [34]. Sequence analysis of 55 *B. napus *lines showed that the *BnA7.SUT1 *gene could be classified into three genotypes. The type III genotype showed 98% genomic sequence similarity with the type II genotype. The polymorphisms were located in the 5'UTR, and there was a 3-bp indel polymorphism at position 229-231 between the type I and type III genotypes; the type II genotype contained either polymorphism. Upstream of the indel, type II and type III genotypes showed the same polymorphisms among the three genotypes; in contrast, downstream of the indel, type I and type II presented the same polymorphisms. Hence, we hypothesized that type II is the result of rearrangements between type I and type III, although this hypothesis requires further molecular evidence. Newly generated chimeras were selected and maintained in a population; however, the *BnA7.SUT1.a- BnA7.SUT1.b *genotype could not be found in association with the promoter of *BnA7.SUT1.a *and may have been selected against during the breeding process. Similar recombination of *HvFT1 *(barley) has generated various alleles [35], which support our findings. The *Zep *allele was generated as a result of recombination with different promoters and regulated the expression level of *Zep *[36].

### Association between *BnA7.SUT1 *and yield-related traits

SUTs drive translocation of sucrose and in turn affect seed yield and fruit size [18,37]. In another study carried out in our laboratory, identification of yield-related QTLs in a *B. napus *functional map [38] indicated that a functional marker from *SUT *in the A7 linkage group was related to EFB, SP, and TSW in the A7 linkage group [31,39]. Here, we report likely polymorphisms in *BnA7.SUT1 *associated with yield-related traits, and allelic variation at the promoter and CDS of *BnA7.SUT1 *correlated with expression pattern and phenotype. Polymorphisms at the promoter and CDS regions with an effect on expression abundance are likely candidates for causative QTPs [40,41]. Similarly, allelic polymorphism at the promoter and intron of *HvFT1 *in barley contribute to variation in flowering time [35].

SUTs have three types of clades designated as protype SUT1 (clade I), SUT2 (clade II), and SUT4 (clade III) [38,42-44], and *BnA7.SUT1 *falls into the protype SUT1 (clade I). Generally, SUT1 mRNA and protein, notably OsSUT1, AtSUC2, and StSUT1, are present in mature phloem and primarily involved in phloem loading. The rice SUT, OsSUT1, plays a significant role in sucrose transport in developing shoots and roots, which is a decisive factor for seed germination and early seedling growth [42]. *AtSUC1 *also has a role in vegetative growth and for normal gametophyte functioning [16]. In higher plants, sugars and hormones interact and form an intricate regulatory sensing and signaling network [45], and altered sucrose levels can change the quantity of sucrose-derived metabolites and sucrose-specific signaling [46]. In the current work, *BnA7.SUT1 *showed higher expression levels in the stem of type II and type III genotypes, consistent with increased EFB. These results indicated that blocking translocation of sucrose at the stem influences either carbon abundance for metabolism or signals.

Oilseed plants with lush branches and leaves, in contrast, produce many empty pods and shrunken seeds at maturity, resulting from insufficient import to the developing seeds [47]. Breeding experiences indicated that translocation of carbohydrate assimilate from source to sink is a major constraint on seed yield [29,48]. The lower expression of *BnA7.SUT1 *in type I genotypes resulted in the lowest yield in the three genotype lines, suggested that retardation of photoassimilate translocation leads to decreased yield. At the pod-filling stage, most leaves abscise and the pod remains primarily a photosynthetic organ [49]. The volume of the pod is estimated at 20 DAF, and the dry weight of the pod reaches a maximum at 25 DAF when sucrose is stored in the pericarp for the later period of seed development [48]. The lower expression observed here of *BnA7.SUT1 *in the pod and developing seed suggests an effective role in the photoassimilate unloading process. *BnA7.SUT1 *showed a reduced expression trend as the pod reached maximum weight (25 DAF). The low expression level of *BnA7.SUT1 *in the pod may be the result of the failure of transportation of sucrose in the developing seed. In our investigation, seed weight was negatively correlated with seed yield. Plants with fewer branches and siliques could distribute more storage substance into each seed with a resulting larger seed weight, indicating that 'sink' is sufficient in general oilseed plants. 'Source' is also not the limitation for seed yield in oilseed [29,30]. Therefore, 'flow' is the most important factor and controls seed yield. Our results support this reasoning. The promoter *BnA7.SUT1.b *correlation with increasing EFB number may be a potential resource for breeding.

Some key genes are closely correlated with yield-related traits. Of great interest, application of dwarfing genes caused a green revolution in the 1960s and doubled grain production only in 40 years [50-52]. On the other hand, most phenotypic variation of agronomic traits is continuous and remarkably influenced by different alleles. In tomato, alleles of *fw2.2 *result in fruit size variation up to 30% and appear to have been responsible for a key transition during domestication [53]. Plant architecture is very important for improving yield traits; *tb1 *acts as a major contributor to apical dominance in maize and regulates lateral branching in rice [54,55]. Moreover, *GS3 *and *Ghd7 *show significant effects on grain size and multiple yield-related traits in rice, respectively [56,57]. Thus, characterization and application of crucial genes/alleles may be an effective means of improving yield. Our investigation indicated that *SUT *may play an important role in sucrose translocation and affect seed yield in *B. napus*. Investigations with larger and/or natural sets of *B. napus *are necessary to validate the association. Furthermore, alteration of sucrose concentration will provide convincing evidence. However, the current characterization of allelic variation and association with yield may be a potential basis for breeding.

## Conclusions

Previous QTL analysis of seed yield-related trait associations with functional markers showed that *SUT *was located in the QTL interval associated with branch number and seed weight. In this study, we isolated different alleles of *BnA7.SUT1 *and identified three genotypes. Lines with the *BnA7.SUT1.b *promoter exhibited better seed yield-related traits. At the pod development phase, *BnA7.SUT1 *showed an increased expression level and a decreasing trend with increasing seed weight. These results indicate reduced transport of photoassimilate from source to sink. *BnA7.SUT1 *may play an important role in photoassimilate accumulation and storage, in turn affecting seed yield.

## Methods

### Plant materials

Two *B. napus *lines 'S-1300' and 'Eagle', showing variation in vegetative and reproductive traits, were grown under field conditions and used to isolate *BnX.SUT1*. 'S-1300' is a Chinese semi-winter self-incompatible line, and 'Eagle' is a Swedish spring line. A panel of 55 semi-winter *B. napus *cultivars/lines maintained at Huazhong Agricultural University, Wuhan, PRC, were used for association in this study. F_1_, F_2_, and BC_1 _populations, resulting from the cross '3715'×'3769', were employed for allelism analysis. The TN DH population, resulting from a cross between 'Tapidor' and 'NY7' [58], served for mapping *BnA7.SUT1*. Marker-differentiated cultivars/lines were planted during 2008-2010 at Huazhong Agricultural University. Their leaves, shoots, flower buds, pods, pericarps of pods, and young seeds at the filling stage (20 DAF), leaves, and pods at different developmental stages were used for expression analysis by real-time PCR.

### Field experiments and trait measurements

The experiments were conducted in three locations (Wuhan, Huanggang, Yichang) in Hubei Province, China. Fifty-five *B. napus *cultivars/lines were grown for two consecutive growing seasons during 2007/08 and 2008/09 at Huazhong Agricultural University, Wuhan, China; they were also grown at the local Academy of Agricultural Science sites in Yichang and Huanggang during 2008/09. Rapeseed plots were subsequently followed by rice crops in all experimental fields. All trials were designed as randomized blocks with three replications in each environment. Each plot consisted of three rows, 3.5 m length with 0.25 m distance between rows. Seeds were sown between the last 5 days of September and the first 5 days of October with the distance between plants in each row reduced to 0.15 cm at 40 days post-emergence. All trials were managed following normal, standard agricultural practices.

At maturity, 12 plants in the middle row were randomly harvested from each plot for evaluation of the following quantitative traits: number of EFB, number of SP, number of SS, TSW, and seed YP. Residual plants of each block were harvested and used to determine BY (Additional file 6).

### DNA extraction and genetic mapping of *BnA7.SUT1*

Genomic DNA of the planted materials, including parents, segregating population, and the panel of 55 cultivars/lines, was extracted from young leaves according to CTAB methodology [59], and DNA from three individuals in each variety/line was mixed for PCR analysis. Based on sequences of the BAC (AC189334) and *BnA7.SUT1*, the SSR marker and gene-specific primers were designed and used to map the gene in the TN DH population using JoinMap 3.0 http://www.kyazma.nl/index.php/mc.JoinMap.

### Sequencing and analysis

Promoter and partial CDS regions of *BnA7.SUT1 *were generated using primer pairs as follows: PT1-L/PT1-R and PT5-L/PT1-R. PCR was performed in reaction volumes of 20 μL containing the following: 50 ng genomic DNA, 1 unit *Taq *polymerase (MBI Fermentas, Lithuania), 2 μL 10× *Taq *buffer with (NH_4_)_2_SO_4_, 2 mM MgCl_2_, 0.2 mM dNTP mix (Sangon, China), and 0.5 μM of each primer. PCR conditions were initial denaturation for 4 min at 94°C, 30 cycles of 45 s at 94°C, annealing at 60°C for 45 s, and extension for 60 s at 72°C, followed by an extension of 10 min at 72°C. PCR products were separated by 1.2% agarose gel electrophoresis and detected by staining with ethidium bromide. The PCR products were purified using the Gel Purification Kit (Sago, Shanghai, China) and ligated into the pMD18-T vector (TaKaRa, Japan). Positive transformed clones were selected for sequencing. The fragments were analyzed using the SEQMAN application of the DNASTAR software suite (Windows version 5.0.2; DNASTAR, Madison, WI, USA) and aligned in MEGA 4.0 [60]. Singletons, which occurred only once as polymorphisms among the sequenced materials, were analyzed until they were confirmed as correct. The LD level between sites and Tajima's *D *statistic were calculated using TASSEL 2.1 [61].

### Population structure and statistical analyses

The AFLP technique, following the protocol of Vos et al. [62] with minor modifications by Lu et al. [63], was employed to genotype the 55 breeding lines. AFLP primers amplify different marker alleles at multiple loci in the allotetraploid *B. napus *genome. It is difficult or impossible to assign the different marker alleles to individual loci in genotypes with high allelic diversity. All AFLP alleles were scored as present or absent in each genotype. In total, 139 polymorphisms were obtained with restriction enzymes *PstI/MseI*. Subsequently, the data were used to infer the population structure (Q) with the model-based Bayesian clustering approach in the software STRUCTURE 2.2 [64]. The membership coefficients were calculated as 10 independent runs for each k (set from 1 to 10) with a burn-in of 50, 000 iterations followed by 50, 000 interactions. A summary of the average of data likelihoods (*LnP(D)*) is shown in Additional file 7.

Mean values, variance components of each yield-related trait, heritability, and correlation coefficients were calculated, respectively. Variance components were computed for lines, environments, interaction between lines and environments, and error. Broad-sense heritability was estimated according to the formula $h^{2}=\sigma_{\text{g}}^{2}∕\left(\sigma_{\text{g}}^{2}+\sigma_{\text{gl}}^{2}∕n+\sigma_{\text{e}}^{2}∕nr\right)$, where $\sigma_{\text{g}}^{2}$ is the genotypic variance, $\sigma_{\text{gl}}^{2}$ is the interactional variance of genotype and environment, $\sigma_{\text{e}}^{2}$ is the error variance, *r *is the number of replicates of each environment, and *n *is the number of environments [65].

Associations between polymorphism sites and yield-related traits were implemented using general linear model analysis in the software package TASSEL 2.1 [61]. The Q matrix was used as the covariate in the analysis to control the population structure. All polymorphisms were tested, and *P *values for individual polymorphisms were estimated based on 10, 000 permutations. The rescaled *P *value accounts for the proportion of the random marker with a permuted *P *value less than or equal to 0.05. Data from each test environment were calculated independently.

### RNA extraction and real-time PCR

Total RNAs were extracted from respective tissues using Tripure reagent (Bioteke, http://www.bioteke.com/chn/). Subsequently, the cDNAs were synthesized with M-MLV reverse transcriptase and an oligo (dT) primer (Fermentas, USA) in a 20 μL volume according to the manufacturer's instructions. The resultant first strand cDNA mixture was diluted with sterile distilled water and used as a template for PCR and for real-time PCR. Real-time quantitative PCR was performed using the SYBR Green Realtime PCR Master Mix (TOYOBO, Osaka, Japan). The PCR reactions contained 400 nM of both forward and reverse gene-specific primers and 8.4 μL of the 50-fold diluted reverse transcriptase (RT) reaction in a final volume of 20 μL. The thermal cycling protocol was followed by DNA polymerase activation at 95°C for 3 min. The PCR amplification was carried out with 45 cycles of denaturation at 95°C for 10 s, primer annealing at 60°C for 15 s, and extension at 72°C for 30 s. Optical data were acquired following the extension step, and the PCR reactions were subject to melting curve analysis beginning at 65°C through 95°C, at 0.1°C s^-1^. The amplified products were sequenced to ensure that each primer pair amplified one specific gene. The data are presented as an average ± SD of three independently produced RT preparations used for PCR runs, each having four replicates. The relative expression levels were calculated using the 2^-ΔΔC^_T _method [66].

## Authors' contributions

FL designed and carried out the linkage, association, and expression analyses and wrote the initial draft of the manuscript. CM conceived of and supervised the overall research. XW participated in the sequence amplification and alignment. CG, JZ, YW, and NC participated in field experimentation. XL implemented field management. JW, BY, JS, JT, and TF helped draft the manuscript. All authors have read and approved the final manuscript.

## Supplementary Material

Additional file 1 **Sequence of primers used to isolate *BnA7.SUT1 *and analyze the expression pattern of *BnA7.SUT1***. M1-M4 were used to generate the main genomic segments of *BnA7.SUT1*; STAs and STBs were used to generate promoter sequences of *BnA7.SUT1.a *and *BnA7.SUT1.b *with degenerate primers for TAD1-6, respectively; RT-A and RT-B were used to analyze the *BnA7.SUT1 *expression pattern, with AC (derived from *B. napus Actin*, GeneBank accession number: AF111812) as an endogenous control. Click here for file

Additional file 2 **Comparison of 44 cDNA sequences isolated from various organs/tissue**. Click here for file

Additional file 3 **An un-rooted tree was developed using the ClustalX program based on available amino acid sequences of SUTs**. Sucrose transporters (SUTs) are from *Asarina barclaiana*: AbSUT1 (AAF04294); *Apium graveolens*: AgSUT3 (ABB89051); *Alonsoa meridionalis*: AmSUT1 (AAF04295); *Arabidopsis thaliana*: AtSUC1 (CAA53147), AtSUC2 (CAA53150), AtSUC3 (AAC32907), AtSUC4 (NP_172467), AtSUC5 (AAG52226), AtSUC6 (NP_199174), AtSUC7 (NP_176830), AtSUC8 (NP_179074), AtSUC9 (NP_196235), *Brssica napus*: BnSUTx (ACB47398); *Brassica oleracea*: BoSUC1 (AAL58071), BoSUC2 (AAL58072); *Bambusa oldhamii*: BoSUT1 (AAY43226); *Citrus sinensis*: CsSUT2 (AAM29153); *Datisca glomerata*: DgSUT4 (CAG70682); *Daucus carota*: DcSUT1 (BAA89458); *Euphorbia esula*: EeSUCx (AAF65765); *Eucommia ulmoides*: EuSUT2 (AAX49396); *Hevea brasiliensis*: HbSUT2a (ABJ51934), HbSUT2b (ABJ51932), HbSUT5 (ABK60189); *Hordeum vulgare*: HvSUT1 (CAB75882), HvSUT2 (CAB75881); *Juglans regia*: JrSUT1 (AAU11810); *Lycopersicum esculentum*: LeSUT2 (AAG12987), LeSUT4 (AAG09270); *Lotus japonicus*: LjSUT4 (CAD61275); *Malus domestica*: MdSUT1 (AAR17700); *Manihot esculenta*: MeSUT2 (ABA08445), MeSUT4 (ABA08443); *Nicotiana tabacum*: NtSUT1 (X82276), NtSUT3 (AAD34610); *Oryza sativa*: OsSUT1 (AAF90181), OsSUT2 (AAN15219), OsSUT3 (BAB68368), OsSUT4 (BAC67164), OsSUT5 (BAC67165); *Plantago major*: PmSUC1 (CAI59556), PmSUC2 (X75764), PmSUC3 (CAD58887); *Populus tremula×Populus tremuloides*: Pt×PtSUT1-1 (CAJ33718); *Pisum sativum*: PsSUT1 (AAD41024); *Ricinus communis*: RcSCR1 (CAA83436); *Solanum demissum*: SdSUT2 (AAT40489); *Saccharum hybridum*: ShSUT1 (AAV41028); *Spinacea oleracea*: SoSUT1 (Q03411); *Solanum tuberosum*: StSUT1 (CAA48915), StSUT4 (AAG25923); *Triticum aestivum*: TaSUT1A (AAM13408), TaSUT1B (AAM13409), TaSUT1D (AAM13410); *Vicia faba*: VfSUCx (CAB07811); *Vitis vinifera*: VvSUCy (AAL32020), VvSUC11 (AAF08329), VvSUC12 (AAF08330), VvSUC27 (AAF08331); *Zea mays*: ZmSUT1 (BAA83501), ZmSUT2 (AAS91375), ZmSUT4 (AAT51689). The BnA7.SUT1 is classified into SUT1 clade. Click here for file

Additional file 4 **A neighbor-joining distance tree for BnA7.SUT1 from different genotype lines**. The numbers on nodes are bootstrap values, and values lower than 60 are not shown. The sequences from *pBnA7.SUT1.a- BnA7.SUT1.a*, *pBnA7.SUT1.b- BnA7.SUT1.a*, and *p BnA7.SUT1.b- BnA7.SUT1.b *form exclusive clades, respectively. Click here for file

Additional file 5 **UPGMA cluster of the panel lines**. Click here for file

Additional file 6 **Phenotypic means for six yield-related traits across four environments**. ^a ^breeding country. Click here for file

Additional file 7 **Summary of the average of the probability of data likelihoods (LnP(D)) for the set of *Brassica napus *genotypes**. Likelihoods were averaged over 10 independent runs with a burn-in of 50, 000 iterations. The set of 55 *Brassica napus *genotypes was tested for K = 1-10 subpopulations. Click here for file
