# Supplementary figures and images for: Characterization of Sucrose transporter alleles and their association with seed yield-related traits in Brassica napus L
Source: BMC Plant Biol. 2011 Nov 23;11:168. doi: 10.1186/1471-2229-11-168 (PMC3248380; doi:10.1186/1471-2229-11-168)

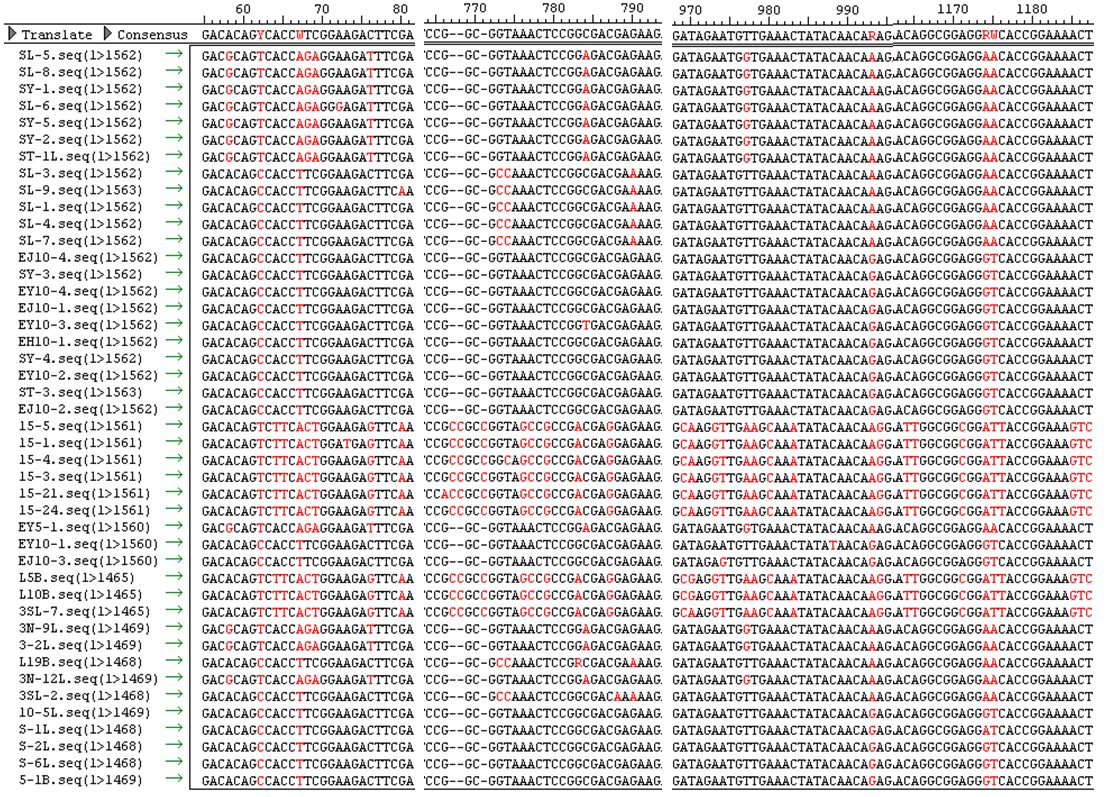

Supplement: Additional file 2 — Comparison of 44 cDNA sequences isolated from various organs/tissue. [file 1471-2229-11-168-S2.BMP]

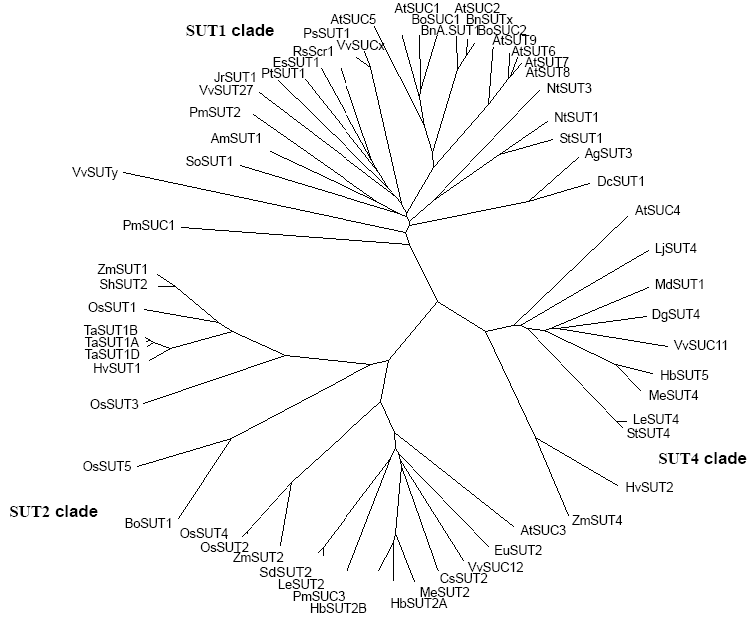

Supplement: Additional file 3 — An un-rooted tree was developed using the ClustalX program based on available amino acid sequences of SUTs. Sucrose transporters (SUTs) are from Asarina barclaiana: AbSUT1 (AAF04294); Apium graveolens: AgSUT3 (ABB89051); Alonsoa meridionalis: AmSUT1 (AAF04295); Arabidopsis thaliana: AtSUC1 (CAA53147), AtSUC2 (CAA53150), AtSUC3 (AAC32907), AtSUC4 (NP_172467), AtSUC5 (AAG52226), AtSUC6 (NP_199174), AtSUC7 (NP_176830), AtSUC8 (NP_179074), AtSUC9 (NP_196235), Brssica napus: BnSUTx (ACB47398); Brassica oleracea: BoSUC1 (AAL58071), BoSUC2 (AAL58072); Bambusa oldhamii: BoSUT1 (AAY43226); Citrus sinensis: CsSUT2 (AAM29153); Datisca glomerata: DgSUT4 (CAG70682); Daucus carota: DcSUT1 (BAA89458); Euphorbia esula: EeSUCx (AAF65765); Eucommia ulmoides: EuSUT2 (AAX49396); Hevea brasiliensis: HbSUT2a (ABJ51934), HbSUT2b (ABJ51932), HbSUT5 (ABK60189); Hordeum vulgare: HvSUT1 (CAB75882), HvSUT2 (CAB75881); Juglans regia: JrSUT1 (AAU11810); Lycopersicum esculentum: LeSUT2 (AAG12987), LeSUT4 (AAG09270); Lotus japonicus: LjSUT4 (CAD61275); Malus domestica: MdSUT1 (AAR17700); Manihot esculenta: MeSUT2 (ABA08445), MeSUT4 (ABA08443); Nicotiana tabacum: NtSUT1 (X82276), NtSUT3 (AAD34610); Oryza sativa: OsSUT1 (AAF90181), OsSUT2 (AAN15219), OsSUT3 (BAB68368), OsSUT4 (BAC67164), OsSUT5 (BAC67165); Plantago major: PmSUC1 (CAI59556), PmSUC2 (X75764), PmSUC3 (CAD58887); Populus tremula×Populus tremuloides: Pt×PtSUT1-1 (CAJ33718); Pisum sativum: PsSUT1 (AAD41024); Ricinus communis: RcSCR1 (CAA83436); Solanum demissum: SdSUT2 (AAT40489); Saccharum hybridum: ShSUT1 (AAV41028); Spinacea oleracea: SoSUT1 (Q03411); Solanum tuberosum: StSUT1 (CAA48915), StSUT4 (AAG25923); Triticum aestivum: TaSUT1A (AAM13408), TaSUT1B (AAM13409), TaSUT1D (AAM13410); Vicia faba: VfSUCx (CAB07811); Vitis vinifera: VvSUCy (AAL32020), VvSUC11 (AAF08329), VvSUC12 (AAF08330), VvSUC27 (AAF08331); Zea mays: ZmSUT1 (BAA83501), ZmSUT2 (AAS91375), ZmSUT4 (AAT51689). The BnA7.SUT1 is classified into SUT1 clade. [file 1471-2229-11-168-S3.BMP]
